# Quercetin-loaded mesenchymal stem cell derived extracellular vesicles enhance ovarian function in a cyclophosphamide induced ovarian damage

**DOI:** 10.1186/s13048-025-01838-5

**Published:** 2025-10-27

**Authors:** Zeynep Ece Utkan Korun, Zehra Seda Halbutogullari, Yusufhan Yazir, Selenay Furat, Candan Altuntas, Ahmet Ozturk, Tugba Koldankaya, Serap Mert, Erkut Attar

**Affiliations:** 1 Department of Obstetrics and Gynecology, Faculty of Medicine, Yeditepe University, Istanbul, Türkiye; 2 Department of Stem Cell and Tissue Regeneration, Institute of Health Sciences, Kocaeli University, Kocaeli, Türkiye; 3 Center for Stem Cell and Gene Therapies Research and Practice, Kocaeli University, Umuttepe West Campus Research Building for Health Sciences, Department of Stem Cell F3, Izmit, Kocaeli, 41001 Türkiye; 4 Department of Medical Biology, Faculty of Medicine, Kocaeli University, Kocaeli, Türkiye; 5 Department of Histology and Embryology, Faculty of Medicine, Kocaeli University, Kocaeli, Türkiye; 6 Department of Polymer Science and Technology, Kocaeli University, Kocaeli, Türkiye; 7 Department of Chemistry, Kocaeli University, Kocaeli, Türkiye

**Keywords:** Premature ovarian failure, Mesenchymal stem cell-derived extracellular vesicles, Wharton’s jelly, Quercetin, Oxidative stress, Folliculogenesis

## Abstract

**Background:**

To investigate whether intraovarian administration of quercetin-loaded extracellular vesicles derived from Wharton’s jelly mesenchymal stem cells (EVs-QUE) improves ovarian function in a cyclophosphamide (CTX)-induced premature ovarian insufficiency (POI) rat model.

**Methods:**

Human Wharton’s jelly-derived mesenchymal stem cells (MSCs) were cultured, and extracellular vesicles (EVs) were isolated and characterized by flow cytometry and electron microscopy. Quercetin was loaded onto EVs using ultrasonic incubation to generate EVs-QUE. A rat model of CTX-induced POI was established, and the subjects received intraovarian injections of either EVs or EVs-QUE. Ovarian function was assessed through histological evaluation, immunofluorescence staining, and gene expression analysis.

**Results:**

Treatment with EVs-QUE significantly improved ovarian morphology and folliculogenesis, reduced the number of atretic follicles, and decreased Casp3 expression. Proliferation markers (*Ki67*,* Pcna*) and antioxidant genes (*Nrf2*,* Sod1*) were upregulated. Additionally, steroidogenesis- and oocyte-related genes (*Star*,* Gdf9*,* Bmp15*) showed increased expression. Although systemic hormonal alterations were limited, local tissue analysis confirmed a regenerative effect in the EVs-QUE group.

**Conclusions:**

Quercetin-loaded EVs derived from Wharton’s jelly MSCs enhanced ovarian recovery in a CTX-induced POI model through anti-apoptotic, pro-proliferative, and antioxidant pathways. These findings suggest that intraovarian administration of EVs-QUE may represent a promising strategy for fertility preservation and warrant further investigation in long-term and translational studies.

## Background

Premature ovarian insufficiency (POI) is defined as oligo/amenorrhea lasting at least four months in women under 40 years of age, accompanied by a single elevated serum follicle-stimulating hormone (FSH) level greater than 25 IU/L [1]. POI, resulting from ovarian follicle depletion or dysfunction, significantly impairs endocrine and reproductive functions and increases the risk of cardiovascular disease, osteoporosis, infertility, psychological distress, and reduced life expectancy. Approximately 3.5% of women under 40 years are affected by this condition [1].

While POI can be idiopathic, chemotherapy is a primary cause, increasingly relevant due to rising cancer incidence among young women [2]. Cyclophosphamide (CTX) is notably gonadotoxic, causing DNA double-strand breaks in granulosa cells and accelerating ovarian reserve depletion by activating dormant primordial follicles through the PI3K/Akt pathway [3–7]. Hormone replacement therapy (HRT), the standard treatment, alleviates hypoestrogenism symptoms but does not restore ovarian function or fertility, highlighting the urgent need for novel fertility-preserving therapeutic strategies [8, 9].

Recent regenerative medicine approaches, particularly mesenchymal stem cell (MSC) therapies show promise in ovarian rejuvenation [10]. Recent studies have successfully constructed artificial ovaries using decellularized ovarian extracellular matrix seeded with mesenchymal and oogonial stem cells, demonstrating in-vivo folliculogenesis [11]. Wharton’s jelly-derived MSCs (WJ-MSCs) from the human umbilical cord are distinguished by immunomodulatory, paracrine, and regenerative properties and have demonstrated efficacy in animal models of CTX-induced ovarian damage [12–16]. These cells have also been reported to differentiate into oocyte-like cells under specific culture conditions, highlighting their reproductive potential [17].

However, MSC therapies pose risks of immune rejection, tumorigenicity, and ethical challenges [18, 19]. Current evidence suggests MSCs exert therapeutic effects primarily through paracrine signaling mediated by extracellular vesicles (EVs), which transfer bioactive molecules such as proteins, lipids, and nucleic acids to target cells [20–22]. EVs represent a promising, safer cell-free alternative for regenerative therapy. EVs are nanoscale biological carriers characterized by their biocompatibility, low immunogenicity, and natural targeting capacity, which makes them highly attractive as drug delivery systems. By shielding therapeutic molecules from enzymatic degradation and crossing biological barriers such as the blood–brain barrier, EVs provide unique therapeutic advantages [23].

Quercetin, a natural flavonoid, exhibits antioxidative, anti-inflammatory, and anti-apoptotic properties by modulating epigenetic mechanisms, inhibiting the formation of reactive oxygen species (ROS), reducing pro-inflammatory cytokines, and supporting DNA repair [24, 25]. However, its poor solubility and limited bioavailability restrict its translation into clinical applications [26–28]. To overcome these limitations, various strategies such as chemical modification, nanoformulations, and particularly extracellular vesicle (EV)-based delivery systems have been investigated. EVs are considered promising carriers due to their inherent biocompatibility, nanoscale size, and ability to protect and deliver bioactive compounds to target tissues [29] Quercetin-loaded exosomes have been shown to improve cognitive function by exerting neuroprotective effects in Alzheimer’s disease models [30] In another study, engineered EVs loaded with quercetin nanoparticles demonstrated antiviral and anti-inflammatory potential against SARS-CoV-2 [31] Furthermore, Zhuang et al. developed a quercetin-loaded exosome–SPION complex that specifically targeted pancreatic β-cells, underscoring the feasibility of organ-specific delivery [32].

Several techniques have been developed to incorporate therapeutic agents into extracellular vesicles (EVs). Among the most commonly used are incubation, sonication, electroporation, freeze–thaw cycles, and extrusion [23, 33]. Incubation relies on the passive diffusion of compounds across the lipid bilayer, a process particularly effective for hydrophobic agents. In contrast, methods such as sonication, electroporation, freeze–thaw cycles, or extrusion transiently perturb the EV membrane to enhance drug encapsulation efficiency. Although these approaches improve loading capacity (e.g., sonication can reach efficiencies of ~ 30%), they may also compromise vesicle integrity [23]. Among these methods, sonication is widely applied for incorporating small molecules into EVs. This technique employs sound wave–induced shear forces to transiently disrupt the lipid bilayer, thereby facilitating drug entry while maintaining overall vesicle stability [34]. Compared to incubation alone, combining incubation with sonication further enhances loading efficiency and provides better control over drug release. For instance, Kim et al. reported successful encapsulation of paclitaxel and doxorubicin into EVs using a strategy that involved both incubation and sonication for cancer therapy [23]. Similarly, Qi et al. demonstrated that quercetin-loaded exosomes accumulated in the brain and exerted neuroprotective effects in an Alzheimer’s disease model, highlighting the therapeutic promise of this delivery strategy [30].

This study aims to evaluate the therapeutic potential of WJ-MSC-derived EVs in a CTX-induced POI model to determine whether quercetin loading enhances the antioxidative and anti-inflammatory properties of EVs, thereby optimizing their therapeutic efficacy for ovarian function restoration.

## Methods

### WJ-MSC cell culture

Human Wharton’s Jelly-derived mesenchymal stem cells (hWJ-MSCs) and dermal fibroblasts were obtained from STEMBIO Cord Blood, Cell and Tissue Center (TUBITAK Martek, Gebze, Türkiye). Cells were cultured in Dulbecco’s Modified Eagle Medium/Nutrient Mixture F-12 (DMEM-F12), supplemented with 10% fetal bovine serum (FBS) and 1% penicillin-streptomycin, maintained at 37 °C in a humidified 5% CO_2_ atmosphere. Cultures were passaged at 70–80% confluency.

Phenotypic characterization of hWJ-MSCs was performed by flow cytometry using antibodies against CD44, CD73, CD90, CD105 and negative markers CD34, CD45, HLA-DR, with appropriate isotype controls (IgG1 and IgG1/G2a). Flow cytometry data were analyzed using BD Cell Quest™ software.

Multipotency was confirmed by adipogenic and osteogenic differentiation using lineage-specific media (Gibco™ StemPro™ Adipogenesis and Osteogenesis Differentiation Kits). Differentiation was initiated at 50–60% confluency for adipogenesis and 80–90% confluency for osteogenesis. Media were refreshed every three days, and differentiation was evaluated after three weeks. Lipid droplets in adipogenic differentiation were visualized by Oil Red O staining, while osteogenic differentiation was confirmed by Alizarin Red S staining for calcium deposition [35].

### WJ-MSC EV isolation, characterization, and quantification

#### EV isolation

Extracellular vesicles (EV) were isolated from hWJ-MSC-conditioned media supplemented with 1% protease inhibitor cocktail (Pierce Biotechnology, Illinois, USA), using a differential centrifugation protocol as described previously [36]. EV pellets were resuspended in phosphate-buffered saline (PBS, ~ 100–1000 µl) and stored in low-protein-binding microtubes (Thermo Scientific, USA). Protein content was measured by a bicinchoninic acid (BCA) assay (Boster, CA, USA).

#### EV characterization with magnetic beads

EVs were characterized using an immuno-affinity bead-assisted flow cytometry approach. Briefly, EV samples were incubated overnight at 4 °C with 2.7 μm Dynabeads™ magnetic beads (Invitrogen; Thermo Fisher Scientific, Vilnius, Lithuania) coated with antibodies specific for CD9, CD63, and CD81. This binding step increases the effective particle size of EVs, enabling detection by conventional flow cytometry. Following incubation, bead–EV complexes were stained for 45 min at room temperature with phycoerythrin (PE)-conjugated monoclonal antibodies (BD Biosciences, CA, USA) targeting the corresponding surface markers. After gentle washing to remove unbound antibodies, samples were analyzed on a FACSCalibur flow cytometer (BD Biosciences, San Jose, CA, USA) [37].

#### Dynamic light scattering (DLS) analysis

Dynamic light scattering (DLS) analysis was used for particle size measurement. EV samples were diluted 1:100 in PBS and analysed with Zetasizer Nano ZS90 (v7.01, Malvern, UK). Three independent measurements per sample were taken, and the mean particle size was calculated [36].

#### Scanning electron microscopy (SEM) analysis

Morphological characterization was conducted using an environmental scanning electron microscope (ESEM, Quattro S, Thermo Fisher Scientific, US). Briefly, 5 µL aliquot of each sample was air-dried on a grids and examined with a STEM 3 detector [36].

#### Quercetin loading and HPLC analysis

Quercetin loading into EVs was performed by ultrasound incubation. Briefly, 1 mg quercetin (Santa Cruz Biotechnology, Texas, USA) was dissolved in 200 µL DMSO containing 2% Tween-80 and mixed with 1.5 mg EVs. The mixture was incubated under continuous shaking at 4 °C overnight (~ 16 h), followed by sonication in an ice-water bath (Alex Machine AXUY-08LAB) with a frequency of 32 kHz ± 5 kHz to enhance cargo incorporation while minimizing thermal and shear stress [29, 30]. Unencapsulated quercetin was removed by ultrafiltration using centrifugal filter units with a molecular weight cut-off (MWCO) of 100 kDa (Corning, NY, USA) [38].

Quercetin loading was quantified by high-performance liquid chromatography (HPLC) using a 1260 Infinity system (Agilent Technologies, USA) equipped with a ZORBAX SB-C18 column (4.6 × 150 mm, 3.5 μm). Data were acquired and analyzed with Agilent ChemStation software. The mobile phase consisted of acetonitrile and water (60:40), with a flow rate of 1 ml/min, injection volume of 20 µL, and detection at 256 nm wavelength. Quercetin retention time was 8 min. Encapsulation efficiency (EE) of quercetin in EVs was calculated as follows [22, 39]:

$$\begin{aligned}\mathrm{EE}\left(\%\right)=&[\left(\text{Initial drug amount}-\right. \\& \left. \text{Free drug amount in supernatant}\right)\\& /\text{Initial drug amount}]\mathrm{x}\,100\end{aligned}$$

#### DiR staining for visualizing cellular uptake

To visualize cellular uptake and localization, EVs were labelled with the lipophilic fluorescent dye DiR [DiIC18(7) (1,1’-Dioctadecyl-3,3,3’,3’-Tetramethylindotricarbocyanine Iodide)] fluorophore (AAT Bioquest, Pleasanton, CA). Dermal fibroblasts were incubated with DiR-labeled EVs for 30 min at room temperature. After incubation, cells were washed with PBS, fixed, and stained with DAPI for nuclear visualization. Due to quercetin’s autofluorescence, additional imaging channels were utilized for quercetin-loaded EVs (EVs-QUE). Fluorescent images were captured using confocal microscopy (Leica SP8 Lightning, Germany) [40, 41].

### In vivo experimental design

All animal experiments were approved by the Kocaeli University Animal Experiments Local Ethics Committee (approval no. KOÜ HAYDEK 7/3–2020). POI was induced in female rats via intraperitoneal cyclophosphamide (120 mg/kg, twice, one week apart) [39]. Estrous cycles were monitored by vaginal smears 14 days after the final CTX injection to confirm POI induction. Hematoxylin and eosin (H&E) stanning confirmed cycle stages.

Rats were housed under standart conditions (22 ± 2 °C, 12-hour light/dark cycle, lights on at 7:00 a.m.). Procedures, including vaginal smear collection, were conducted between 9:00–11:00 a.m. Animals were randomly assigned to five groups (*n* = 6 per group):

1. Control group (no intervention).
2. POI group (CTX-induced POI, treated with PBS).
3. EVs group (CTX-induced POI, treated with hWJ-MSC-derived EVs).
4. QUE group (CTX-induced POI, treated with quercetin).
5. EVs-QUE group (CTX-induced POI, treated with quercetin-loaded EVs).

Under anesthesia (ketamine/xylazine), ovaries were exposed via a dorsal incision. Treatments (10 µg protein/ovary) were administered intraovarian at two cortical regions using a Hamilton syringe (5 µL per site). Quercetin dose was matched to EVs-QUE. Incisions were sutured, and the procedure was repeated on the contralateral ovary. One ovary per rat was stored in RNAlater^®^ (RNA Stabilization Solution, Thermo Fisher Scientific) for quantitative real-time PCR (qRT-PCR) analysis, while the contralateral ovary was fixed in 10% neutral buffered formalin for histological and immunocytochemical evaluations.

### Histological analysis and follicle counting

Formalin-fixed ovarian tissues were embedded in paraffin and sectioned at 5 μm thickness. Every fifth section was selected and stained with H&E. Follicles with visible oocyte nuclei were counted, and the total follicle number was estimated by multiplying the counted follicles by five. Follicles were classified into developmental stages as primordial, primary, secondary, Graafian follicles, corpus luteum, and atretic follicles according to previously established criteria [42].

### Immunofluorescence staining and confocal microscopy

Paraffin-embedded ovarian sections were deparaffinized, rehydrated, and permeabilized using PBS containing Triton X-100. Non-specific binding sites were blocked with an appropriate blocking solution. Sections were incubated overnight at 4 °C with primary antibodies against Caspase-3 (1:250, ab92552, Abcam, Cambridge, UK) and Pcna (1:250, ab92552, Abcam), both diluted in PBS. After washing with PBS, slides were incubated for 1 h at room temperature with fluorescently labeled secondary antibodies (1:1000, ab150083 for Pcna, Abcam). Nuclei were counterstained using DAPI-containing mounting medium (UltraCruz, Texas, USA), and coverslips were applied. Fluorescent images were captured with confocal laser scanning microscope (Leica SP8 Lightning, Germany). Positive staining was quantified using ImageJ Software.

### Enzyme-Linked immunosorbent assay (ELISA)

Serum levels of follicle-stimulating hormone (FSH) and anti-Müllerian hormone (AMH) were measured using commercially available ELISA kits (Cusabio, Houston, TX, USA). Assays were performed on 96-well plates pre-coated with specific antibodies, following the manufacturer’s instructions. Absorbance was read at 450 nm using a microplate reader (Molecular Devices, Versamax microplate reader, USA).

### Gene expression analysis by qRT-PCR

Total RNA was extracted from ovarian tissues using a commercial RNA isolation kit, and reverse-transcribed into complementary DNA (cDNA) using the High-Capacity cDNA Reverse Transcription Kit (Thermo Fisher Scientific, CA, USA). Quantitative real-time PCR (qRT-PCR) was performed using AMPIGENE qPCR Green Mix (Enzo Biochem, USA) and gene-specific primers listed in Table 1. Reactions were run on a LightCycler^®^ 480 system (Roche, Germany).

**Table 1:** Primer sequences used for the amplification of target genes

Gene Name	Accesion Number	Primer Sequences Forward (F)- Revers (*R*)
Follicle-stimulating hormone receptor (*Fshr*)	NM_199237	F: CATTCTTGGGCACGGGATCT R: GGTGAGCACAAACCTCAGTTC
Anti-Mullerian hormone receptor type 2 (*Amhr2*)	NM_030998	F: TTCCAAGGAAGCGTTGACGA R: TCCTGTTTGGGGATACTTGTGC
Stem cell factor (*Kitl*)	NM_021843	F: AGCAGTAGCAGTAATAGGAAAGCC R: GTGCCATTGCTGTCCATTGT
Steroidogenic acute regulatory protein (*Star*)	NM_031558	F: GAAGAACTGGTGGACCGCAT R: TCAGGCATCTCCCCAAAGTG
Bone morphogenetic protein 15 (*Bmp15*)	NM_021670	F: TCGGGTTCTGCAAAGCCTTCT R: TCCCTTGCGATTCCAGAGCTT
Growth differentiation factor-9 (*Gdf9*)	NM_021672	F: CTGTTGGGGGTTTGCTGCTT R: TTAGGGGTCTCACTTCGCCT
NFE2 like bZIP transcription factor 2 (*Nrf2*)	NM_031789	F: GTCAGCTACTCCCAGGTTGC R: CCAAACTTGCTCCATGTCCT
Superoxide dismutase 1 (*Sod1*)	NM_017050	F: CGGATGAAGAGAGGCATGTT R: CACTTTGCCCAAGTCATCT
Marker of proliferation Ki-67 (*Ki67*)	NM_001271366	F: GCAGACAAGCCTTCAGCAGTAA R: TGGTACCATTGTCATCAATTTCAGT
Caspase 3 (*Casp3*)	NM_012922	F: GTGGAACTGACGATGATATGGC R: CGCAAAGTGACTGGATGAACC

Targeted genes included markers related to folliculogenesis (*Fshr*,* Amhr2*,* Gdf9*,* Bmp15*,* Kitl)*, steroidogenesis (*Star*), proliferation (*Ki67*), apoptosis (*Casp3*), and oxidative stress (*Nrf2*,* Sod1*). Expression levels were normalized to the reference gene *Actb*. Fold changes were calculated using the 2^−ΔΔCt^ method.

### Statistical analysis

Statistical analyses were conducted using IBM SPSS Statistics, version 27 (IBM Corp., Armonk, NY, USA). Depending on data distribution, either Student’s t-test or Kruskal-Wallis one-way ANOVA was applied, followed by appropriate post hoc testing where applicable. Results are presented as mean ± SD or SEM, as indicated in figure legends. Statistical significance was set at *p* ≤ 0.05. Asterisks indicate significance levels: *p ≤ 0.05, **p < 0.01, ***p < 0.001.

## Results

### Mesenchymal characteristics and lineage differentiation of hWJ-MSCs, with confirmed expression of EV markers

**Fig. 1:**
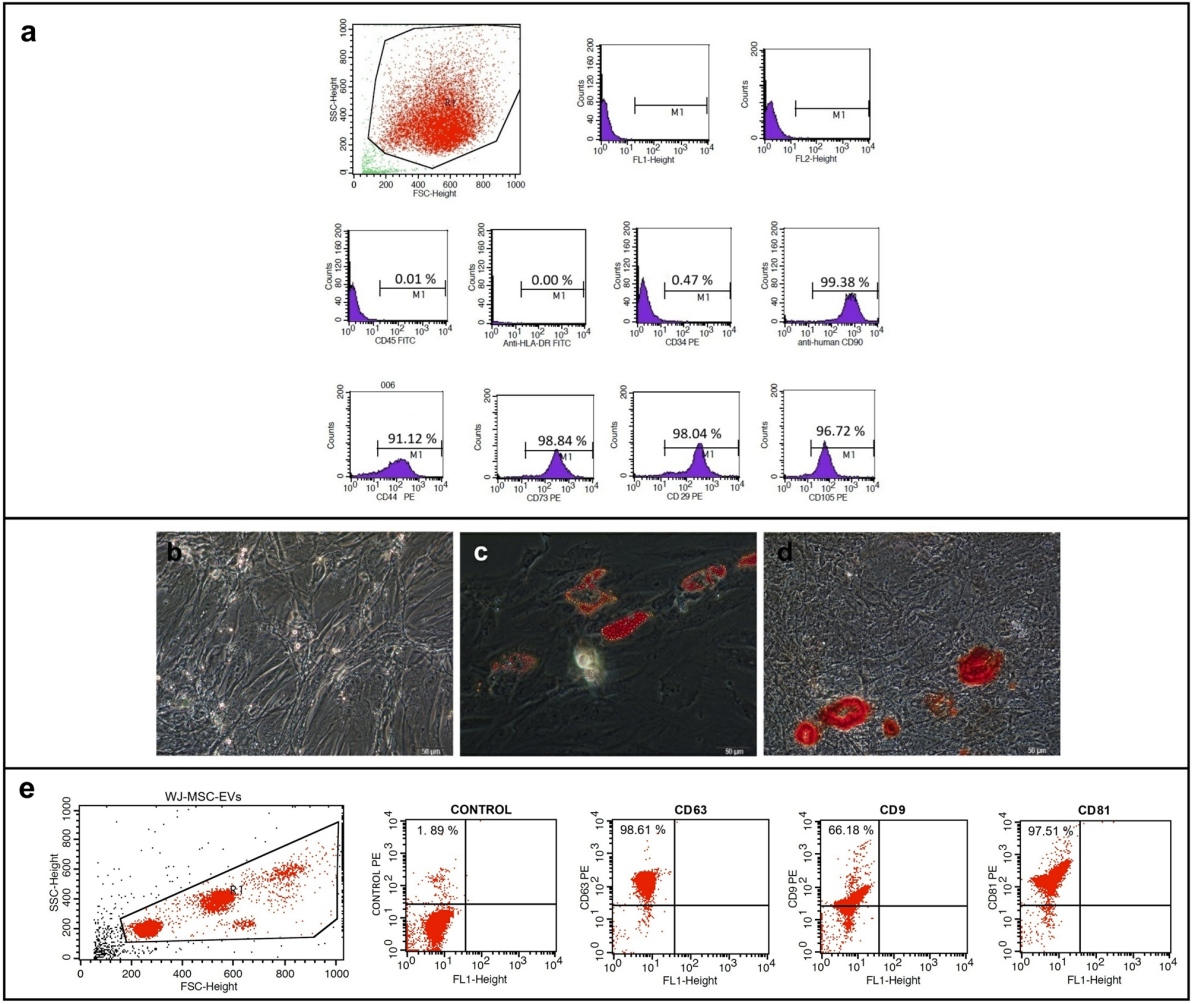
Characterization of human Wharton’s jelly mesenchymal stem cells (hWJ-MSCs): **a** flow cytometry analysis of cells, **b** control group of differentiation, **c** adipogenic differentiation group’s oil red O image by phase-contrast microscope (**d**) osteogenic differentiated group’s alizarin red S image by phase-contrast microscope. Measurement bar: 50 μm, **e** flow cytometry dot-blot analysis of EVs: CD63, CD9, and CD81 EVs markers

hWJ-MSCs expressed mesenchymal markers (CD29, CD44, CD73, CD90, CD105) and lacked hematopoietic markers (HLA-DR, CD34, CD45) (Fig. 1a). Multipotency was validated by lineage-specific differentiation: lipid droplets following adipogenic induction (Oil Red O staining, Fig. 1c) and calcium deposition after osteogenic differentiation (Alizarin Red S staining, Fig. 1d). No staining was observed in undifferentiated controls (Fig.S1b). EVs, isolated from hWJ-MSCs were positive for exosomal markers CD9, CD63, and CD81, as shown by flow cytometry (Fig. 1e).

### Quercetin-Loaded EVs exhibit structural integrity and cellular uptake

Quercetin loading into EVs was quantified via HPLC using a standard curve generated with serial dilutions (31.25, 62.5, 125, 250, and 500 µg/mL). Under optimized conditions (acetonitrile/water 60:40; 1 mL/min flow rate; detection at 256 nm), quercetin showed a retention time of ~ 8 min. The encapsulation efficiency was approximately 30–40%.

### Electron microscopy and particle size analysis confirmed successful quercetin loading into EVs

**Fig. 2:**
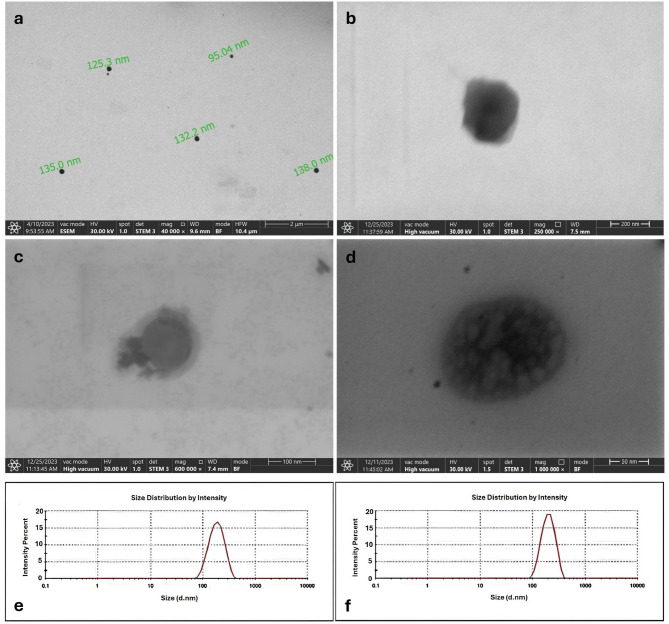
Electron microscopy images zetasizer measurements of extracellular vesicles before and after quercetin loading. **a**, **b** Electron microscopic images of EVs, and (**c**, **d**) EVs-QUEs at different magnifications. **e** Zetasizer measurements of EVs, and (**f**) EVs-QUE's

EVs exhibited spherical morphology with smooth membranes and a uniform size distribution ranging from ~ 95 to 140 nm (Fig. 2a–b). Following quercetin loading, EM images revealed that quercetin was either adsorbed onto the EV surface (Fig. 2c) or internalized (Fig. 2d), while the overall membrane integrity of the vesicles remained preserved. Consistent with this observation, DLS analysis showed a slight increase in average particle size after loading, indicating successful encapsulation and potential surface interaction of quercetin with the vesicles. The mean particle size of EVs-QUE increase to 222.76 ± 49.68 nm (Fig. 2f) while the EVs revealed a mean particle diameter of 169.45 ± 31.45 nm (Fig. 2e).

**Fig. 3:**
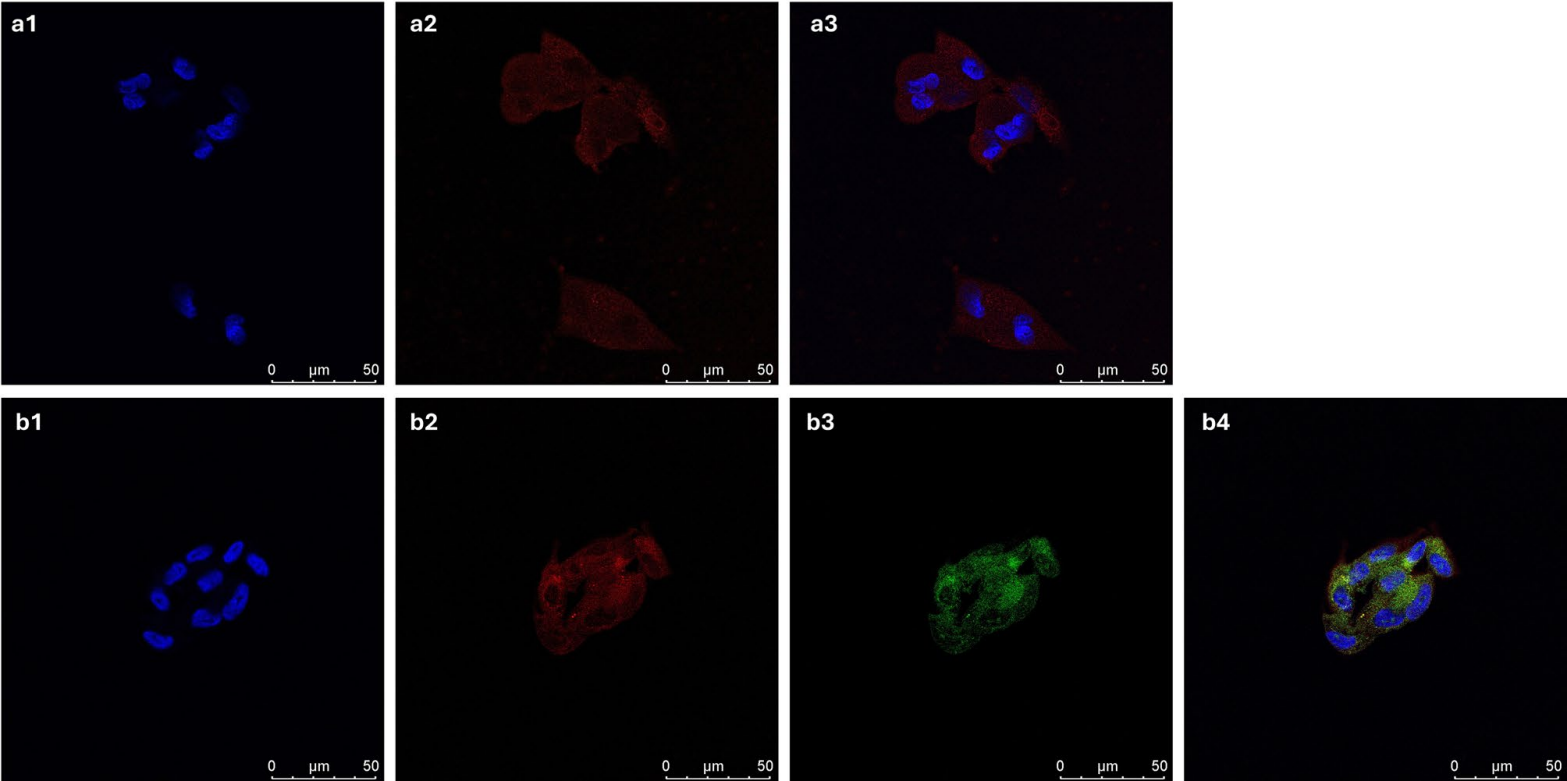
Representative images of DIR iodide stained EVs and EVs-QUE in human dermal fibroblasts at the 12-h time point. **a1**, **b1** DAPI, **a2**, **b2** DİR (red), **b3 **Quercetin (green), and **a3**, **b4** images were superimposed (merge). Measurement bar: 50 μm

Confocal microscopy confirmed the cellular uptake of DiR-labeled EVs. Red fluorescence corresponding to DiR was clearly localized within the cytoplasm, indicating successful internalization of the labeled EVs by the recipient cells (Fig. 3a2, b2). In EVs-QUE treated cells, green autofluorescence of quercetin was observed alongside DiR signal (Fig. 3b3), confirming the co-delivery of EVs and QUE. Overlay images revealed cytoplasmic colocalization of DiR and QUE signals, supporting efficient intracellular delivery (Fig. 3a3, b4).

### Disrupted estrous cycles indicate ovarian dysfunction in the POI model

**Fig. 4:**
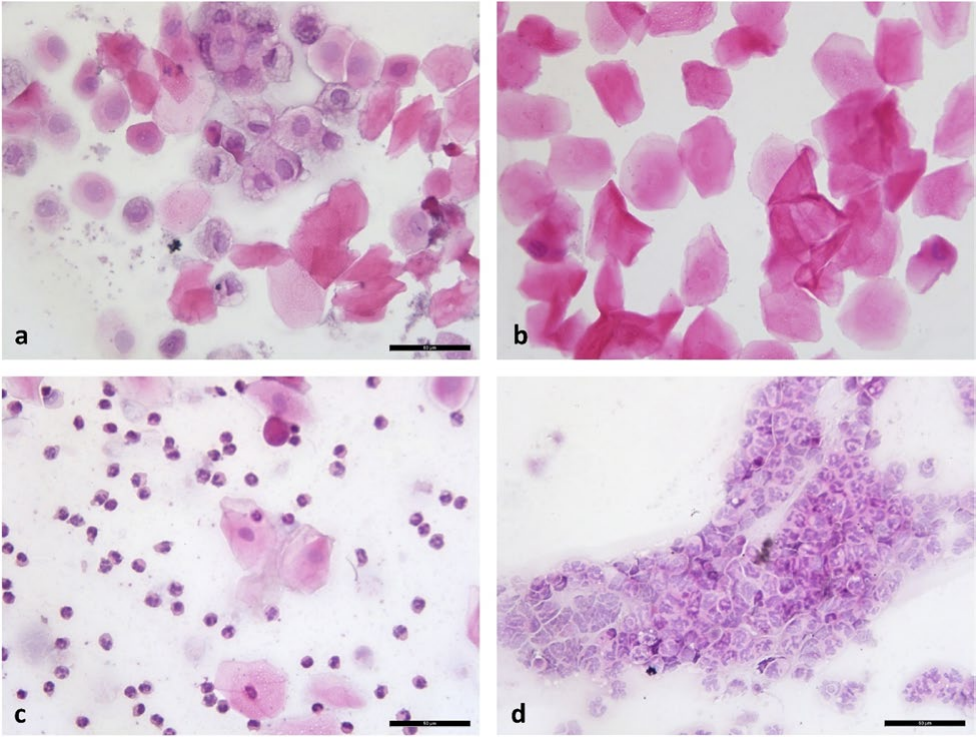
H&E staining photomicrographs of vaginal smears from rats. **a** Proestrus. **b** Estrus. **c** Metestrus. **d** Diestrus. 400x magnification

In control rats, vaginal smear analysis revealed a regular estrous cycle with distinct stages: proestrus was marked by nucleated epithelial cells, estrus by anucleated cornified cells, metestrus by a combination of cornified cells and leukocytes, and diestrus predominantly by leukocytes. (Fig. 4). In contrast, the POI group exhibited irregular and prolonged cycles, indicating impaired ovarian function. The day with the highest number of nucleated epithelial cells was defined as day 1, in accordance with established criteria [43].

The average duration of the estrus cycle was significantly prolonged in the POI group compared to the control group (10.25 ± 1.28 vs. 4.53 ± 0.78 days). In parallel, the total follicle count was markedly reduced in POI animals (79.17 ± 6.46) versus controls (135.52 ± 6.11), whereas the number of atretic follicles was substantially higher in the POI group (29.16 ± 5.16) than in controls (3.83 ± 1.03), indicating impaired folliculogenesis and disrupted ovarian function (Table 2) [43].

**Table 2:** Evaluation of ovarian function between control and POI group. Data are presented as mean ± standard deviation (mean ± SD), *n* = 6 per group. Statistical analysis: (*** p < *0.001*)

Group	Days of estrus cycle	Total follicule count	Atretic follicule count
Control	4.53 ± 0.78	135.52 ± 6.11	3.83 ± 1.03
POI	10.25 ± 1.28 ***	79.17 ± 6.46 ***	29.16 ± 5.16 ***

### EVs-QUE treatment enhances follicular development and reduces ovarian damage in POI rats

**Fig. 5:**
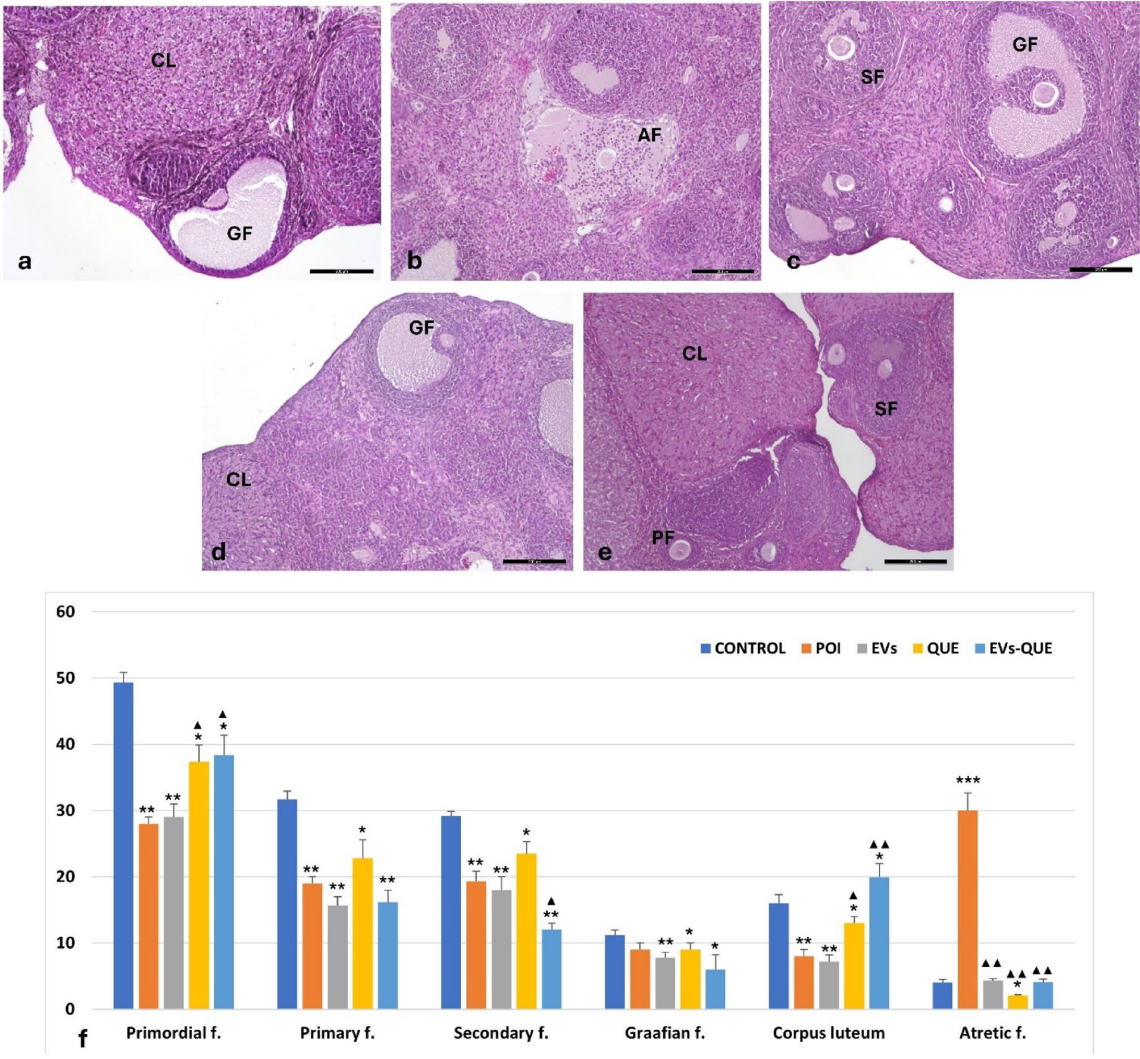
Representative immunhistochemical staining with H&E. Primary follicule (PF), secondary folicule (SF), Graffian follicule (GF), corpus luteum (CL), atretic follicule (AF) (**a**) control, (**b**) POI, (**c**) EVs, (**d**) QUE, (**e**) EVs-QUE groups, (**f**) Quantitative analysis of follicle counts by type (primordial, primary, secondary, Graafian, corpus luteum, and atretic follicles). Data are presented as mean ± standard deviation (mean ± SD), *n* = 6 per group. Statistical analysis: * *p* < 0.05, ** *p <* 0.01, ★★★ *p* < 0.001 vs. Control group; ▲ *p* < 0.05, ▲▲ *p* < 0.01 vs. POI group

H&E staining demonstrated clear differences in follicular morphology among the experimental groups. Ovaries in the control group exhibited normal architecture with healthy primordial, primary, secondary, and Graafian follicles, along with well-developed corpora lutea (Fig. 5a). In contrast, the POI group showed markedly reduced follicle numbers, extensive atresia, and disrupted follicular structures (Fig. 5b). Treatment with EVs or quercetin alone partially restored follicular morphology, as evidenced by the presence of growing follicles, although tissue organization remained less defined compared with controls (Fig. 5c–d). Notably, the combined EVs–QUE treatment preserved ovarian morphology most effectively, with increased numbers of primordial and developing follicles and visible corpora lutea, suggesting enhanced folliculogenesis (Fig. 5e).

The bar graph (Fig. 5f) summarizes the quantitative follicle count analysis across groups. Compared to the control group, the POI group exhibited significantly lower numbers of primordial, primary, secondary, and Graafian follicles (* *p* < 0.05, *** *p* < 0.01) and a marked increase in atretic follicles (*** *p* < 0.001). Compared to the POI group, primordial follicle counts were significantly elevated in the QUE and EVs-QUE groups (▲*p* < 0.05), and the number of corpora lutea was higher in both the QUE (▲*p* < 0.05) and EVs-QUE (▲▲*p* < 0.01) groups. Importantly, the number of atretic follicles was significantly reduced in all treatment groups compared to POI (▲▲*p* < 0.01).

### EVs-QUE treatment reduces apoptosis and enhances cell proliferation in POI ovaries

**Fig. 6:**
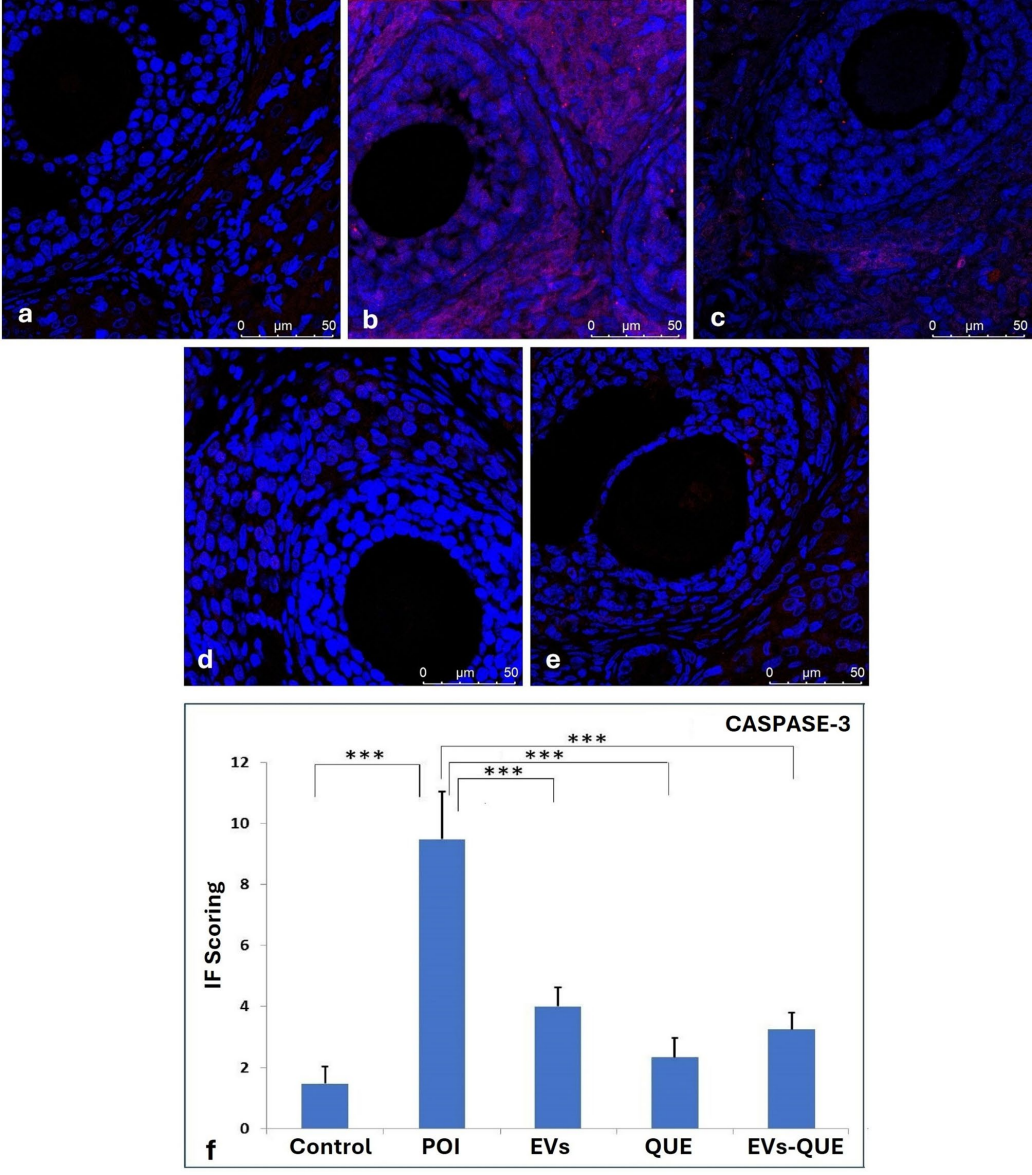
CASPASE-3 immunofluorescence imaging and IF scoring. **a** control, **b** POI, **c** EVs, **d** QUE, **e** EVs-QUE groups, and **f** IF score of experimental groups (***p < 0,001)

Immunofluorescence analysis showed significantly elevated *Caspase-3* expression in the POI group compared to the controls (***p < 0.001), indicating increased apoptosis. Treatment with EVs, QUE and EVs-QUE significantly reduced *Caspase-3* levels, with the most prominent reduction in EVs-QUE group (***p < 0.001) (Fig. 6).

**Fig. 7:**
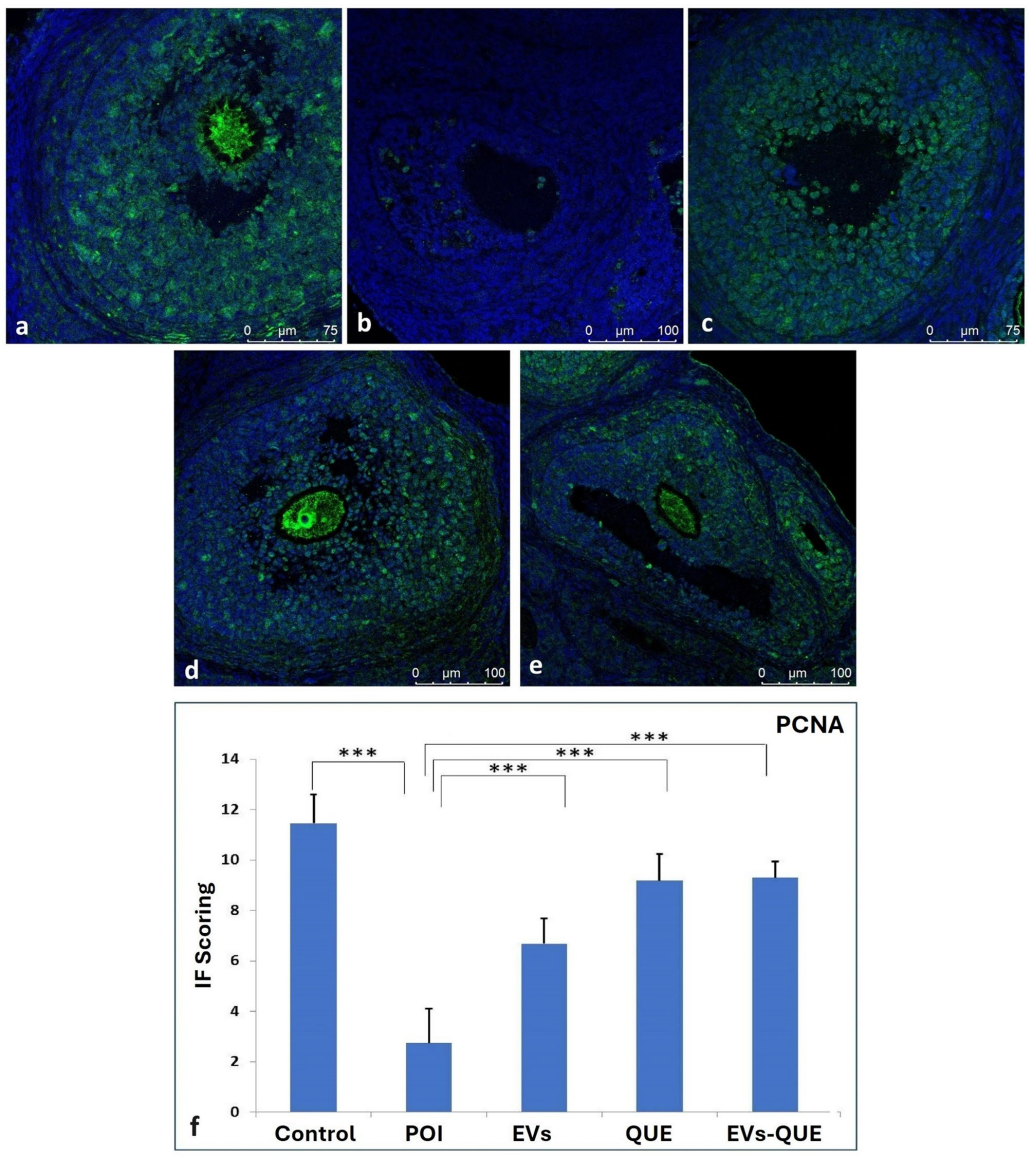
PCNA immunofluorescence image and IF scoring. **a** control, **b** POI, **c** EVs, **d** QUE, **e** EVs-QUE groups, and **f** IF score of experimental groups (***p < 0,001).

*Pcna* expression, a proliferation marker, was markedly downregulated in the POI group (*** p < 0.001), while all treatment groups showed increased expression compared to POI (*** *p* < 0.001) (Fig. 7).

### EVs-QUE modulates serum FSH and AMH levels in POI rats

**Fig. 8:**
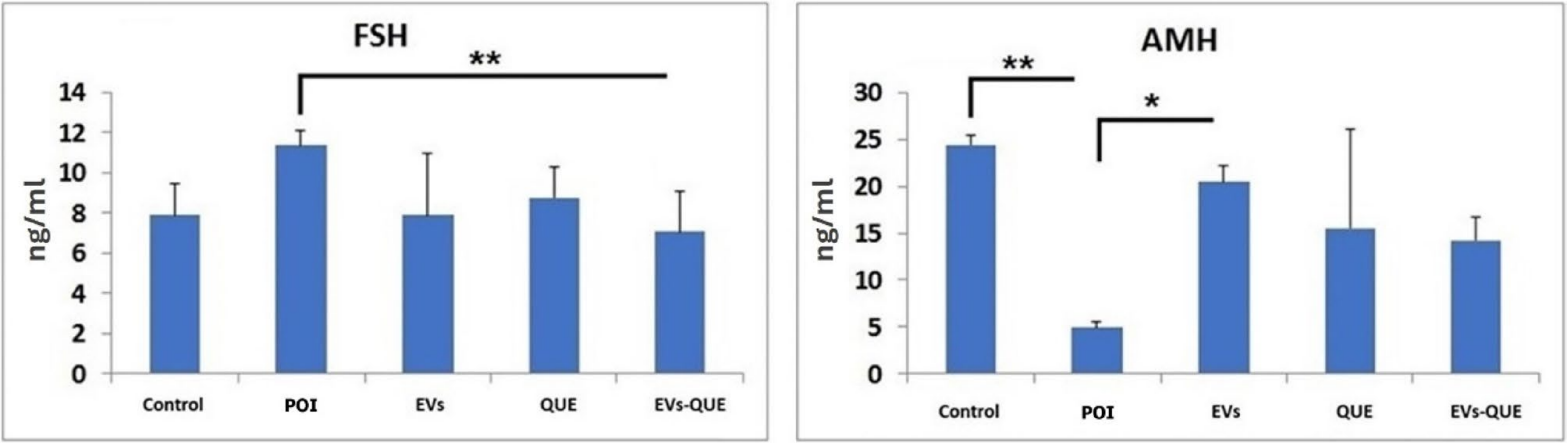
FSH and AMH levels (ng/ml) of ELISA experiment. Values are expressed as mean ± S.E.M. *n* = 6 for each group. (*p < 0.05, **p < 0.01, ***p < 0,001)

Serum FSH levels were significantly decreased in all treatment groups compared to POI, with the most notable reduction in the EVs-QUE group (*p* < 0.01). Fi Similarly, AMH levels increased across all treatment groups. A statistically significant increase in AMH was observed in the EVs group compared to POI (*p* ≤ 0.05) (Fig. 8).

### Gene expression analysis reveals enhanced oocyte and antioxidant pathway activation in EVs-QUE Group by EVs and QUE

**Fig. 9:**
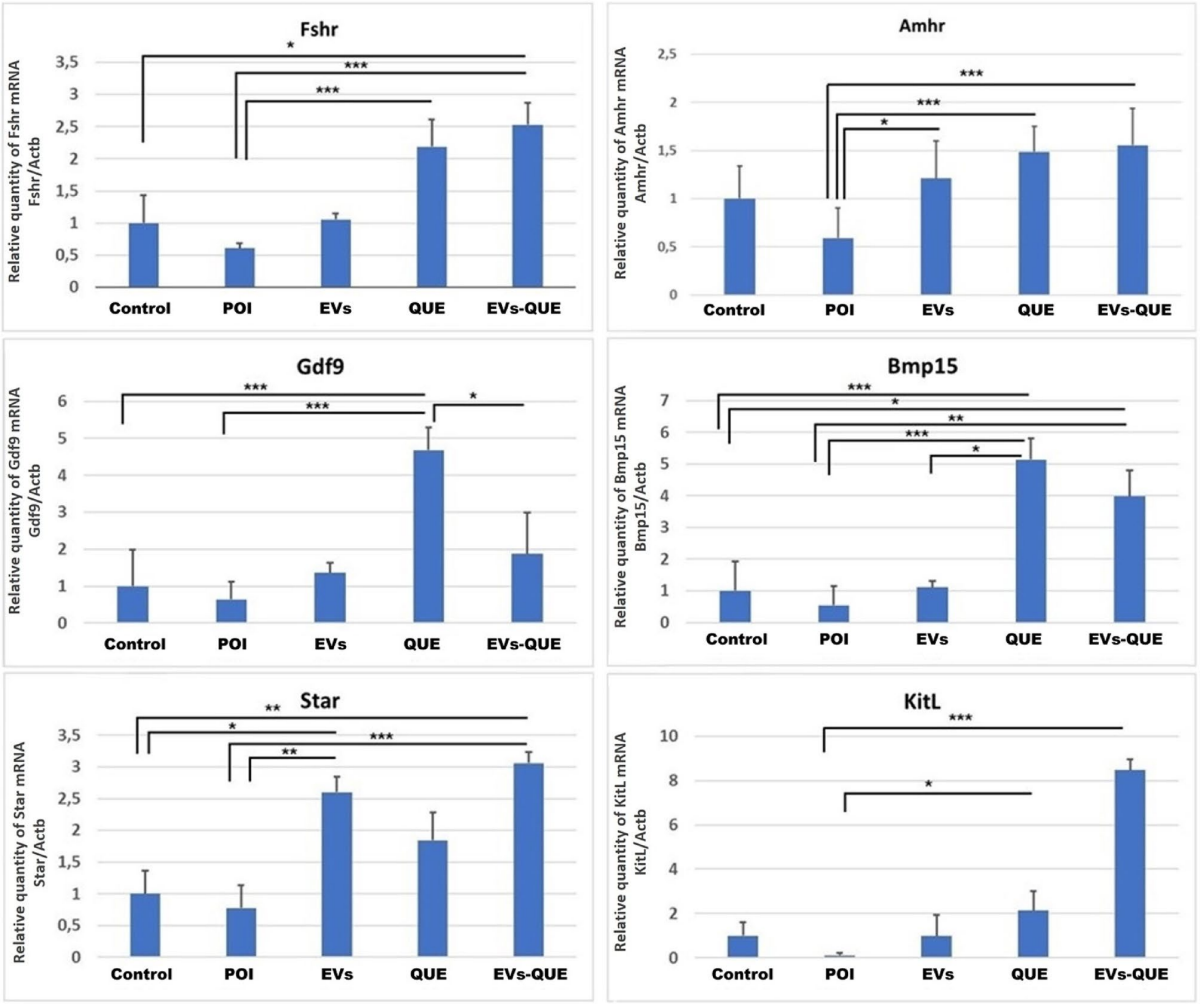
Analysis results of Oocyte related gene expression levels by quantitative real-time-PCR. (X-axis represents the experimental groups, Y-axis represents the fold changes relative to the reference gene *Actb*) Values are expressed as mean ± S.E.M. *n* = 6 for each group. (*p < 0.05, **p < 0.01, ***p < 0,001)

Gene expression analysis revealed significant intergroup differences. *Fshr* was significantly upregulated in the QUE and EVs-QUE groups (*p* < 0,001), but not in the EVs group. *Amhr2* expression was significantly elevated in the EVs (*p* ≤ 0,05), QUE (*p* < 0.001), and EVs-QUE *(p* < 0.001) groups. *Bmp15* was significantly upregulated in the QUE (*p* < 0.001) and EVs-QUE (*p* < 0.01) groups. *Gdf9* expression was significantly increased in the QUE group (*p* < 0.001); although it increased in EVs-QUE group, this was not statistically significant. *Star* expression was elevated in all treatment groups, with significant increases in the EVs (*p* < 0.01) and EVs-QUE (*p* < 0.01) groups. *KitL* was significantly upregulated in the QUE (*p* ≤ 0.05) and EVs-QUE (*p* < 0.001) groups (Fig. 9).

**Fig. 10:**
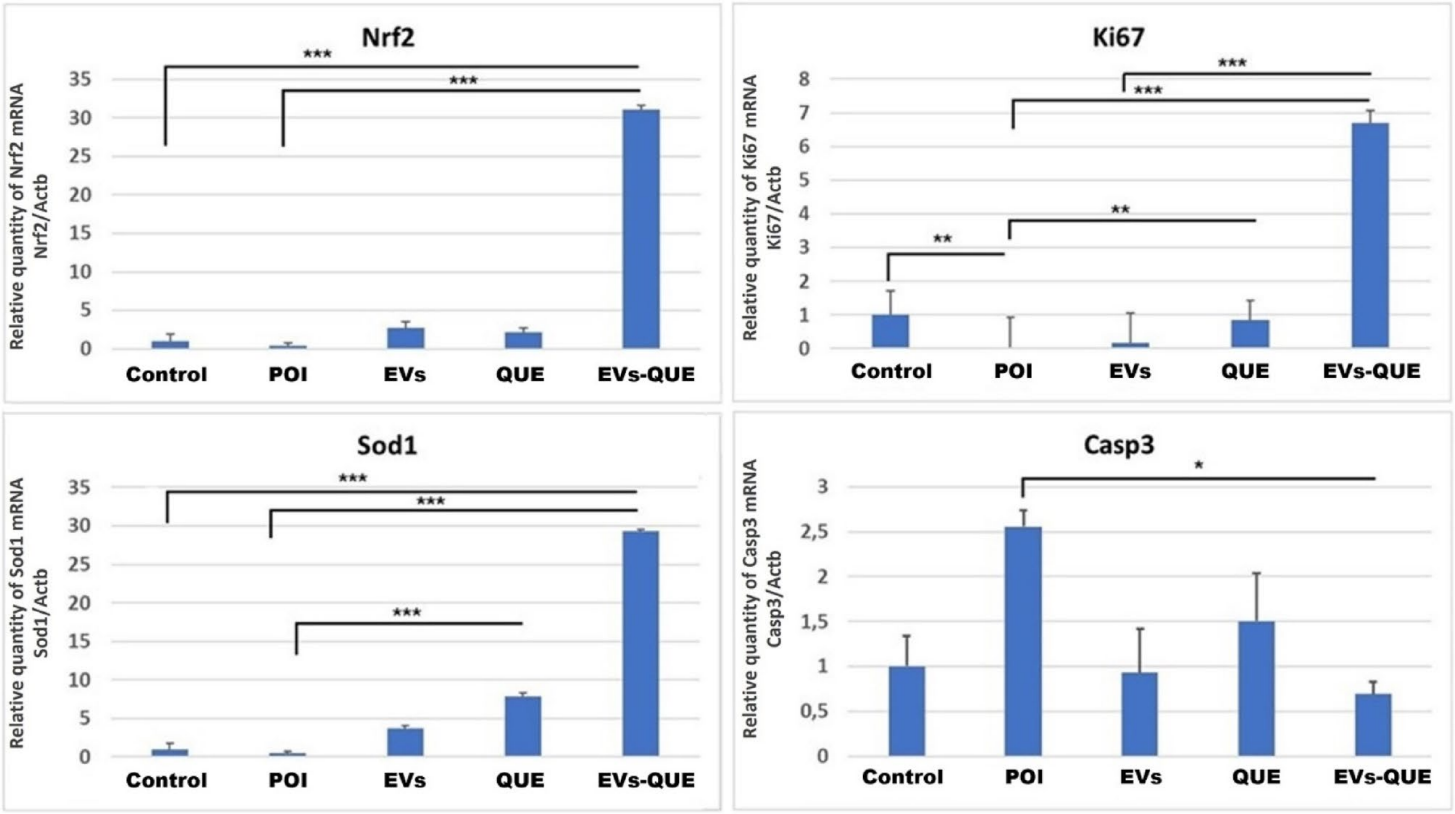
Analysis results of ROS related gene expression levels by quantitative real-time-PCR (X-axis represents the experimental groups, Y-axis represents the fold changes relative to the reference gene *Actb*) Values are expressed as mean ± S.E.M. *n* = 6 for each group. (*p < 0.05, **p < 0.01, ***p < 0,001)

In oxidative stress and apoptosis-related genes, *Nrf2* was markedly elevated in the EVs-QUE group (*p* < 0.001). *Ki67* expression was significantly lower in the POI group (*p* < 0.01) and significantly higher in the QUE (*p* < 0,01) and EVs-QUE (*p* < 0.001) groups. Notably, *Ki67* levels in the EVs-QUE group were also significantly higher than in the EVs group (*p* < 0.001). *Sod1* was significantly upregulated in both the QUE and EVs-QUE groups (*p* < 0.001). *Casp3* expression, was reduced in all treatment groups compared to the POI group, with a statistically significant decrease observed only in the EVs-QUE group (*p* ≤ 0.05) (Fig. 10).

## Discussion

This study is the first to evaluate the therapeutic effects of quercetin-loaded extracellular vesicles derived from Wharton’s jelly mesenchymal stem cells (EVs-QUE) in a cyclophosphamide-induced premature ovarian insufficiency model. Given the limited treatment options for preserving fertility in POI, this approach offers a promising new avenue. The results demonstrated that EVs-QUE enhanced ovarian function through multiple mechanisms, including improved folliculogenesis, reduced apoptosis, increased cellular proliferation, and mitigation of oxidative stress. Upregulation of antioxidant genes (*Nrf2*,* Sod1*) and the proliferation marker *Ki67*, along with downregulation of *Casp3*, were observed. Additionally, elevated expression of *Gdf9*,* Bmp15*, and *Star* suggested support for oocyte maturation and steroidogenesis. These findings indicate that EVs-QUE may offer a promising strategy for ovarian function restoration.

POI represents a significant clinical challenge with limited treatment options. Although HRT improves symptoms of hypoestrogenism, it fails to restore ovarian reserve or fertility and may carry risks such as thromboembolism and malignancy [44, 45]. Consequently, alternative strategies are needed to preserve or restore reproductive capacity.

Recent advances in regenerative medicine, particularly the application of mesenchymal stem cell-based therapies, have shown promise in reversing ovarian damage. MSCs derived from bone marrow, amniotic fluid, adipose tissue, and umbilical cord have been extensively studied in both preclinical and clinical settings [14, 46–48]. Among them, Wharton’s jelly-derived MSCs are notable for their low immunogenicity and ethical acceptability [16–50]. Studies have showned that umbilical cord-derived MSCs can improve hormonal profiles and promote folliculogenesis in chemotherapy-induced ovarian injury models [2, 14, 51].

However, direct MSC therapy faces several challenges, including immune rejection, tumorigenic potential, and difficulties in cell delivery. Increasing evidence indicates that MSCs exert their therapeutic effects primarily via paracrine mechanisms, notably through extracellular vesicles [20, 45, 52]. EVs, with their ability to carry bioactive molecules and mimic parental cell function, provide a cell-free alternative with improved safety and scalability.

Quercetin, a dietary flavonoid with established antioxidative, anti-inflammatory, and anti-apoptotic properties, has been investigated in various models of oxidative stress-related damage. It modulates gene expression by enhancing antioxidant pathways (*Prx1-6*, *Nrf2*) and suppressing inflammatory mediators such as *IL-1β*,* COX-2*, and *iNOS* [47]. In CTX-induced ovarian injury models, both oral and intraperitoneal administration of quercetin has been shown to enhance folliculogenesis, reduce follicular atresia, and preserve granulosa cell structure [53–55]. However, the underlying mechanisms remain incompletely understood. Importantly, to the best of our knowledge, no previous study has evaluated the direct intraovarian administration of quercetin, underscoring the novelty and translational relevance of the current approach. In line with this, our findings from the QUE group demonstrated significant improvements in ovarian morphology and function, supporting the therapeutic potential of localized quercetin delivery.

Despite its potential, quercetin is limited by poor solubility and stability. To overcome these challenges, delivery systems such as nanoparticles have been explored. Extracellular vesicles offer a biocompatible, cell-free platform with natural targeting capacity [56]. For instance, Qi et al. demonstrated that quercetin-loaded exosomes accumulated in the cerebellum and exerted neuroprotective effects in an Alzheimer’s model [30]. Moreover, several studies have highlighted that EV-mediated delivery improves the bioavailability, cellular uptake, and therapeutic efficacy of quercetin compared to its free form. Zhuang et al. showed that quercetin-loaded SPION-exosome complexes preserved pancreatic β-cell survival and function in a rat model of type 2 diabetes by reducing ROS and inflammatory signaling [32]. Similarly, in a COVID-19-related lung injury model, quercetin-loaded EVs modulated macrophage polarization and suppressed cytokine release more effectively than free quercetin [31].

In this study, both EVs and EVs-QUE were injected directly into the ovarian cortex. Although technically demanding, this local administration strategy may enhance tissue-specific effects while minimizing systemic exposure. Fluorescent labeling confirmed successful uptake of EVs by dermal fibroblasts in vitro. In accordance with previous literature, intraovarian injection of stem cells or their derivates has been associated with reduced ovarian apoptosis [57, 58].

Zhang et al. demonstrated that intraovarian administration of exosomes derived from menstrual blood stromal cells promoted follicle development, increased granulosa cell proliferation, and reduced apoptosis in POI model. These changes were supported by histological and hormonal improvements, along with upregulation of K*i67* and downregulation of apoptotic genes *Bax* and *Casp8* [43]. Similarly, Park et al. reported that intraovarian injection of human umbilical cord-derived MSC-EVs was superior to systemic administration in restoring ovarian function in an experimental PCOS model [59]. Other studies have also supported the therapeutic benefits of locally delivered EVs. Sun et al. showed that hUMSC-EVs upregulated *Bcl2* and downregulated *Bax* and *Caspase-3* in granulosa cells [60]. Ding et al. demonstrated that intraovarian exosome injection suppressed *Sirt7*, a key oxidative stress regulator, while Xiao et al. found that amniotic fluid-derived exosomes carrying miR-10a and miR-146a decreased ROS accumulation and inhibited apoptotic signaling [15, 44]. In line with these findings, our study uniquely combined intraovarian delivery with a bioactive cargo-quercetin.

Our gene expression analysis revealed that all treatment groups—EVs, QUE, and EVs–QUE—contributed to the restoration of ovarian function in the POI model. While EVs and QUE alone improved folliculogenesis, reduced follicular atresia, and modulated genes related to oxidative stress and oocyte development, the EVs–QUE group produced the most consistent effects, including marked downregulation of *Casp3* and significant upregulation of *Nrf2*,* Sod1*,* Star*,* Gdf9*,* Bmp15*, and *Kitl*, supporting enhanced antioxidative defense, proliferation, and steroidogenic activity. These findings are consistent with previous reports showing the importance of *Gdf9* and *Bmp15* in folliculogenesis [61] and with studies by Hu et al. and Noda et al., which highlighted the protective roles of *Nrf2* and *Sod1* in oxidative stress regulation [62, 63]. Collectively, these results confirm that while both EVs and quercetin individually exert therapeutic benefits through paracrine signaling and antioxidative/anti-apoptotic pathways, encapsulation of quercetin within EVs provides superior intracellular delivery and bioavailability. Together, these data underscore the complementary potential of EVs, QUE, and their combination, with EVs–QUE representing the most effective strategy for promoting ovarian tissue regeneration.

Immunofluorescence analysis further supported the regenerative impact of all treatment groups. While the POI group showed increased Caspase3 expression, all treatment groups demonstrated a reduction. Similarly, Pcna expression, which was suppressed in the POI group was significantly upregulated following treatment, indicating active tissue repair. These histological and molecular findings complement the gene expression data, highlighting the anti-apoptotic and pro-proliferative effects of the therapy.

Unlike systemic delivery routes, the intraovarian approach used in this study may have resulted in more localized biological effects, which could explain the modest hormonal changes observed. Among treatment groups, only the EVs–QUE group showed a significant decrease in serum FSH levels, while AMH was significantly elevated in the EVs group. Follicle counts also revealed greater increases in primordial follicles and corpus luteum formation, particularly with EVs–QUE treatment. These findings are consistent with previous reports demonstrating that direct intraovarian or paracrine-based therapies improve folliculogenesis and partially restore hormonal balance [64, 65]. Taken together, our data support the therapeutic potential of intraovarian administration of quercetin-loaded EVs as a targeted strategy for ovarian tissue regeneration, suggesting that EVs may enhance quercetin’s effects by improving its bioavailability, stability, and intracellular delivery.

We acknowledge, however, the limitation of not including a co-administration group (EVs + free QUE), which would have provided additional insights into potential additive versus synergistic effects. This aspect has been noted as a limitation and future investigations incorporating such groups will be necessary to further clarify the specific contribution of EV-mediated encapsulation.

## Conclusion

This study demonstrates that quercetin-loaded extracellular vesicles derived from Wharton’s jelly mesenchymal stem cells (EVs-QUE) significantly enhance ovarian recovery in a cyclophosphamide-induced premature ovarian insufficiency model. EVs-QUE improved folliculogenesis, reduced apoptosis, promoted cellular proliferation, and alleviated oxidative stress. These effects were supported by histological, molecular, and gene expression findings. Importantly, the direct intraovarian delivery of EVs-QUE enabled targeted tissue regeneration with potentially reduced systemic effects. This cell-free, localized therapeutic strategy holds promise for fertility preservation in patients at risk of chemotherapy-induced ovarian failure. Further studies are needed to evaluate long-term reproductive outcomes, offspring health, and translational feasibility.

## Data Availability

The data underlying this article will be shared upon reasonable request to the corresponding author.

## References

[1] Panay N, Anderson RA, Bennie A, Cedars M, Davies M, Ee C, et al. Evidence-based guideline: premature ovarian insufficiency. Hum Reprod Open. 2024;2024:hoae065. 39660328 10.1093/hropen/hoae065 PMC11631070

[2] Song D, Zhong Y, Qian C, Zou Q, Ou J, Shi Y, et al. Human umbilical cord mesenchymal stem cells therapy in cyclophosphamide-induced premature ovarian failure rat model. BioMed Res Int. 2016;2016:2517514. 27047962 10.1155/2016/2517514 PMC4800076

[3] Letourneau JM, Ebbel EE, Katz PP, Oktay KH, McCulloch CE, Ai WZ, et al. Acute ovarian failure underestimates age-specific reproductive impairment for young women undergoing chemotherapy for cancer. Cancer. 2012;118:1933–9. 21850728 10.1002/cncr.26403 PMC3220922

[4] Miller JJ III, Williams GF, Leissring JC. Multiple late complications of therapy with cyclophosphamide, including ovarian destruction. Am J Med. 1971;50:530–5. 5313910 10.1016/0002-9343(71)90341-x

[5] Plowchalk DR, Meadows MJ, Mattison DR. Reproductive toxicity of cyclophosphamide in the C57BL/6 N mouse: 2. Effects on uterine structure and function. Reprod Toxicol. 1992;6:423–9. 1463922 10.1016/0890-6238(92)90005-e

[6] Ben-Aharon I, Shalgi R. What lies behind chemotherapy-induced ovarian toxicity? Reproduction. 2012;144:153–63. 22653316 10.1530/REP-12-0121

[7] Kalich-Philosoph L, Roness H, Carmely A, Fishel Bartal M, Ligumsky H, Paglin S, et al. Cyclophosphamide triggers follicle activation and Burnout; AS101 prevents follicle loss and preserves fertility. Sci Transl Med. 2013;5:185ra62. 23677591 10.1126/scitranslmed.3005402

[8] Practice C. on G. Hormone therapy in primary ovarian insufficiency. Obstetrics and gynecology. 2017;129:E134–41. 10.1097/AOG.0000000000002044 28426619

[9] Kovanci E, Schutt AK. Premature ovarian failure: clinical presentation and treatment. Obstet Gynecol Clin North Am. 2015;42:153–61. 25681846 10.1016/j.ogc.2014.10.004

[10] Sheikhansari G, Aghebati-Maleki L, Nouri M, Jadidi-Niaragh F, Yousefi M. Current approaches for the treatment of premature ovarian failure with stem cell therapy. Biomed Pharmacother. 2018;102:254–62. 29567538 10.1016/j.biopha.2018.03.056

[11] Mirzaeian L, Eivazkhani F, Saber M, Moini A, Esfandiari F, Valojerdi MR, et al. In-vivo oogenesis of oogonial and mesenchymal stem cells seeded in transplanted ovarian extracellular matrix. J Ovarian Res. 2023;16:56. 10.1186/s13048-023-01131-3. 36941728 10.1186/s13048-023-01131-3 PMC10029222

[12] Takehara Y, Yabuuchi A, Ezoe K, Kuroda T, Yamadera R, Sano C, et al. The restorative effects of adipose-derived mesenchymal stem cells on damaged ovarian function. Lab Invest. 2013;93:181–93. 23212100 10.1038/labinvest.2012.167 PMC3561594

[13] Umer A, Khan N, Greene DL, Habiba UE, Shamim S, Khayam AU. The therapeutic potential of human umbilical cord derived mesenchymal stem cells for the treatment of premature ovarian failure. Stem Cell Rev Rep. 2023;19:651–66. 36520408 10.1007/s12015-022-10493-y PMC10070285

[14] Wang X, Li T, Bai X, Zhu Y, Zhang M, Wang L. Therapeutic prospect on umbilical cord mesenchymal stem cells in animal model with primary ovarian insufficiency: a meta-analysis. Front Med. 2023;10:1211070. 10.3389/fmed.2023.1211070 PMC10264577 37324123

[15] Xiao G-Y, Liu I-H, Cheng C-C, Chang C-C, Lee Y-H, Cheng WT-K, et al. Amniotic fluid stem cells prevent follicle Atresia and rescue fertility of mice with premature ovarian failure induced by chemotherapy. PLoS ONE. 2014;9:e106538. 25198549 10.1371/journal.pone.0106538 PMC4157795

[16] Rady D, Abbass M, El-Rashidy AA, El Moshy S, Radwan IA, Dörfer CE, et al. Mesenchymal stem/progenitor cells: the prospect of human clinical translation. Stem Cells Int. 2020. 10.1155/2020/8837654. 33953753 10.1155/2020/8837654 PMC8063852

[17] Zolfaghar M, Mirzaeian L, Beiki B, Naji T, Moini A, Eftekhari-Yazdi P, et al. Wharton’s jelly derived mesenchymal stem cells differentiate into oocyte like cells *in vitro* by follicular fluid and cumulus cells conditioned medium. Heliyon. 2020. 10.1016/j.heliyon.2020.e04992. 33088934 10.1016/j.heliyon.2020.e04992 PMC7560581

[18] Zhang Q, Sun J, Huang Y, Bu S, Guo Y, Gu T, et al. Human amniotic epithelial cell-derived exosomes restore ovarian function by transferring microRNAs against apoptosis. Mol Ther. 2019;16:407–18. 10.1016/j.omtn.2019.03.008 PMC6479666 31022607

[19] Zhu W, Huang L, Li Y, Qian H, Shan X, Yan Y, et al. Mesenchymal stem cell-secreted soluble signaling molecules potentiate tumor growth. Cell Cycle. 2011;10:3198–207. 10.4161/cc.10.18.17638. 21900753 10.4161/cc.10.18.17638

[20] Horie M, Choi H, Lee RH, Reger RL, Ylostalo J, Muneta T, et al. Intra-articular injection of human mesenchymal stem cells (MSCs) promote rat meniscal regeneration by being activated to express Indian Hedgehog that enhances expression of type II collagen. Osteoarthritis Cartilage. 2012;20:1197–207. 22750747 10.1016/j.joca.2012.06.002 PMC3788634

[21] Pegtel DM, Gould SJ, Exosomes. Annu Rev Biochem. 2019;88:487–514. 31220978 10.1146/annurev-biochem-013118-111902

[22] Su C, Li H, Shi Y, Wang G, Liu L, Zhao L, et al. Carboxymethyl-β-cyclodextrin conjugated nanoparticles facilitate therapy for folate receptor-positive tumor with the mediation of folic acid. Int J Pharm. 2014;474:202–11. 25149123 10.1016/j.ijpharm.2014.08.026

[23] Kim HI, Park J, Zhu Y, Wang X, Han Y, Zhang D. Recent advances in extracellular vesicles for therapeutic cargo delivery. Exp Mol Med. 2024. 10.1038/s12276-024-01201-6. 38556545 10.1038/s12276-024-01201-6 PMC11059217

[24] Shankar S, Kumar D, Srivastava RK. Epigenetic modifications by dietary phytochemicals: implications for personalized nutrition. Pharmacol Ther. 2013. 10.1016/j.pharmthera.2012.11.002. 23159372 10.1016/j.pharmthera.2012.11.002 PMC4153856

[25] Chen JC, Ho FM, Chao PDL, Chen CP, Jeng KCG, Hsu HB, et al. Inhibition of iNOS gene expression by Quercetin is mediated by the Inhibition of IκB kinase, nuclear factor-kappa B and STAT1, and depends on Heme oxygenase-1 induction in mouse BV-2 microglia. Eur J Pharmacol. 2005;521:9–20. 16171798 10.1016/j.ejphar.2005.08.005

[26] Scheepens A, Tan K, Paxton JW. Improving the oral bioavailability of beneficial polyphenols through designed synergies. Genes Nutr. 2010. 10.1007/s12263-009-0148-z. 19841960 10.1007/s12263-009-0148-z PMC2820202

[27] Terao J. Factors modulating bioavailability of quercetin-related flavonoids and the consequences of their vascular function. Biochem Pharmacol. 2017;139:15–23. 10.1016/j.bcp.2017.03.021 28377278

[28] Alizadeh SR, Ebrahimzadeh MA, Elsevier Masson s.r.l. Quercetin derivatives: drug design, development, and biological activities, a review. Eur J Med Chem. 2022. 10.1016/j.ejmech.2021.114068. 34971873 10.1016/j.ejmech.2021.114068

[29] Yang C, Xu T, Lu Y, Liu J, Chen C, Wang H, et al. Quercetin-loaded human umbilical cord mesenchymal stem cell-derived sevs for spinal cord injury recovery. Neuroscience. 2024;552:14–28. 38806069 10.1016/j.neuroscience.2024.05.028

[30] Qi Y, Guo L, Jiang Y, Shi Y, Sui H, Zhao L. Brain delivery of quercetin-loaded exosomes improved cognitive function in AD mice by inhibiting phosphorylated tau-mediated neurofibrillary tangles. Drug Deliv. 2020;27:745–55. 32397764 10.1080/10717544.2020.1762262 PMC7269046

[31] Raghav A, Giri R, Agarwal S, Kala S, Jeong GB. Protective role of engineered extracellular vesicles loaded Quercetin nanoparticles as anti-viral therapy against SARS-CoV-2 infection: a prospective review. Front Immunol. 2022. 10.3389/fimmu.2022.1040027. 36569877 10.3389/fimmu.2022.1040027 PMC9773252

[32] Zhuang M, Rao L, Chen Y, Xiao S, Xia H, Yang J, et al. Controlled SPION-Exosomes loaded with Quercetin preserves pancreatic beta cell survival and function in type 2 diabetes mellitus. Int J Nanomed. 2023;18:5733–48. 10.2147/IJN.S422416 PMC10578181 37849640

[33] Chen Z, Xiong M, Tian J, Song D, Duan S, Zhang L. Encapsulation and assessment of therapeutic cargo in engineered exosomes: a systematic review. J Nanobiotechnol. 2024. 10.1186/s12951-023-02259-6. 10.1186/s12951-023-02259-6 PMC10765779 38172932

[34] Haney MJ, Klyachko NL, Zhao Y, Gupta R, Plotnikova EG, He Z, et al. Exosomes as drug delivery vehicles for Parkinson’s disease therapy. J Control Release. 2015;207:18–30. 25836593 10.1016/j.jconrel.2015.03.033 PMC4430381

[35] Lennon DP, Schluchter MD, Caplan AI. The effect of extended first passage culture on the proliferation and differentiation of human marrow-derived mesenchymal stem cells. Stem Cells Transl Med. 2012;1:279–88. 23197807 10.5966/sctm.2011-0011 PMC3659698

[36] Öztürk C, Halbutoğullari ZS. Immune regulation is more effective in the U937 inflammation model with mesenchymal stem cell extracellular vesicles stimulated by pro-inflammatory cytokines. Cent Eur J Immunol. 2024;49:282–99. 39720277 10.5114/ceji.2024.143726 PMC11664804

[37] Sovunjov E, Halbutoğulları ZS, Gacar G, Öztürk A, Duruksu G, Yazır Y. Examining the effect of activated cytotoxic (CD8+) T-cell exosomes to the lung cancer. Med Oncol. 2023;40:359. 37966661 10.1007/s12032-023-02198-0

[38] Wu L, Wang L, Liu X, Bai Y, Wu R, Li X, et al. Milk-derived exosomes exhibit versatile effects for improved oral drug delivery. Acta Pharm Sin B. 2022;12:2029–42. 35847507 10.1016/j.apsb.2021.12.015 PMC9279706

[39] Zhang T, Yan DW, Yang Y, Ma A, Li L, Wang Z et al. The comparison of animal models for premature ovarian failure established by several different source of inducers. Regul Toxicol Pharmacol. 2016;81:223–32. Available from: https://api.semanticscholar.org/CorpusID:40744612 10.1016/j.yrtph.2016.09.002 27612992

[40] Mukai R, Shirai Y, Saito N, Yoshida K, Ashida H. Subcellular localization of flavonol aglycone in hepatocytes visualized by confocal laser scanning fluorescence microscope. Cytotechnology. 2009;59:177–82. 19568944 10.1007/s10616-009-9206-z PMC2774565

[41] Santelices J, Ou M, Hui WW, Maegawa GHB, Edelmann MJ. Fluorescent labeling of small extracellular vesicles (EVs) isolated from conditioned media. Bio Protoc. 2022;12:e4447–4447. 35864901 10.21769/BioProtoc.4447 PMC9257841

[42] Chen Y, Zhao Y, Miao C, Yang L, Wang R, Chen B, et al. Quercetin alleviates cyclophosphamide-induced premature ovarian insufficiency in mice by reducing mitochondrial oxidative stress and pyroptosis in granulosa cells. J Ovarian Res. 2022. 10.1186/s13048-022-01080-3. 36572950 10.1186/s13048-022-01080-3 PMC9793602

[43] Zhang X, Zhang L, Li Y, Yin Z, Feng Y, Ji Y. Human umbilical cord mesenchymal stem cells (hUCMSCs) promotes the recovery of ovarian function in a rat model of premature ovarian failure (POF). Gynecol Endocrinol. 2021;37:353–7. 33491494 10.1080/09513590.2021.1878133

[44] Ding C, Zhu L, Shen H, Lu J, Zou Q, Huang C, et al. Exosomal miRNA-17-5p derived from human umbilical cord mesenchymal stem cells improves ovarian function in premature ovarian insufficiency by regulating SIRT7. Stem Cells. 2020;38:1137–48. 32442343 10.1002/stem.3204

[45] Yang Z, Du X, Wang C, Zhang J, Liu C, Li Y, et al. Therapeutic effects of human umbilical cord mesenchymal stem cell-derived microvesicles on premature ovarian insufficiency in mice. Stem Cell Res Ther. 2019;10:1–12. 31412919 10.1186/s13287-019-1327-5 PMC6693188

[46] Bao R, Xu P, Wang Y, Wang J, Xiao L, Li G, et al. Bone marrow derived mesenchymal stem cells transplantation rescues premature ovarian insufficiency induced by chemotherapy. Gynecol Endocrinol. 2018;34:320–6. 29073798 10.1080/09513590.2017.1393661

[47] Fazeli Z, Abedindo A, Omrani MD, Ghaderian SMH. Mesenchymal stem cells (MSCs) therapy for recovery of fertility: a systematic review. Stem Cell Rev Rep. 2018;14:1–12. 28884412 10.1007/s12015-017-9765-x

[48] Wang Z, Wang Y, Yang T, Li J, Yang X. Study of the reparative effects of menstrual-derived stem cells on premature ovarian failure in mice. Stem Cell Res Ther. 2017;8:1–14. 28114977 10.1186/s13287-016-0458-1 PMC5259841

[49] Watson N, Divers R, Kedar R, Mehindru A, Mehindru A, Borlongan MC, et al. Discarded wharton jelly of the human umbilical cord: a viable source for mesenchymal stromal cells. Cytotherapy. 2015;17:18–24. 25442786 10.1016/j.jcyt.2014.08.009 PMC4274214

[50] Yoon SY. Mesenchymal stem cells for restoration of ovarian function. Clin Exp Reprod Med. 2019;46:1. 30827071 10.5653/cerm.2019.46.1.1 PMC6436469

[51] Elfayomy AK, Almasry SM, El-Tarhouny SA, Eldomiaty MA. Human umbilical cord blood-mesenchymal stem cells transplantation renovates the ovarian surface epithelium in a rat model of premature ovarian failure: possible direct and indirect effects. Tissue Cell. 2016;48:370–82. 27233913 10.1016/j.tice.2016.05.001

[52] Hocking AM, Gibran NS. Mesenchymal stem cells: paracrine signaling and differentiation during cutaneous wound repair. Exp Cell Res. 2010;316:2213–9. 20471978 10.1016/j.yexcr.2010.05.009 PMC2902653

[53] Zheng S, Ma M, Chen Y, Li M. Effects of quercetin on ovarian function and regulation of the ovarian PI3K/Akt/FoxO3a signalling pathway and oxidative stress in a rat model of cyclophosphamide-induced premature ovarian failure. Basic Clin Pharmacol Toxicol. 2022;130:240–53. 34841658 10.1111/bcpt.13696

[54] Eren CY, Gurer HG, Gursoy OO, Yilmaz O, Tunc E, Aypak SU, et al. The effect of Quercetin on ovary functions in rats with cyclophosphamide induced ovary damage. Clin Exp Obstet Gynecol. 2024;51:67.

[55] Elkady MA, Shalaby S, Fathi F, El-Mandouh S. Effects of quercetin and rosuvastatin each alone or in combination on cyclophosphamide-induced premature ovarian failure in female albino mice. Hum Exp Toxicol. 2019;38:1283–95. 31370695 10.1177/0960327119865588

[56] Kuo Y-C, Chen C-L, Rajesh R. Optimized liposomes with transactivator of transcription peptide and anti-apoptotic drugs to target hippocampal neurons and prevent tau-hyperphosphorylated neurodegeneration. Acta Biomater. 2019;87:207–22. 30716553 10.1016/j.actbio.2019.01.065

[57] Fu X, He Y, Xie C, Liu W. Bone marrow mesenchymal stem cell transplantation improves ovarian function and structure in rats with chemotherapy-induced ovarian damage. Cytotherapy. 2008;10:353–63. 18574768 10.1080/14653240802035926

[58] Mohamed SA, Shalaby SM, Abdelaziz M, Brakta S, Hill WD, Ismail N, et al. Human mesenchymal stem cells partially reverse infertility in chemotherapy-induced ovarian failure. Reprod Sci. 2018;25:51–63. 28460567 10.1177/1933719117699705 PMC6344979

[59] Park H-S, Cetin E, Siblini H, Seok J, Alkelani H, Alkhrait S, et al. Therapeutic potential of mesenchymal stem cell-derived extracellular vesicles to treat PCOS. Int J Mol Sci. 2023;24:11151. 37446328 10.3390/ijms241311151 PMC10342552

[60] Sun L, Li D, Song K, Wei J, Yao S, Li Z, et al. Exosomes derived from human umbilical cord mesenchymal stem cells protect against cisplatin-induced ovarian granulosa cell stress and apoptosis in vitro. Sci Rep. 2017;7:2552. 28566720 10.1038/s41598-017-02786-x PMC5451424

[61] Sanfins A, Rodrigues P, Albertini DF. GDF-9 and BMP-15 direct the follicle symphony. J Assist Reprod Genet. 2018;35:1741–50. 30039232 10.1007/s10815-018-1268-4 PMC6150895

[62] Hu X, Roberts JR, Apopa PL, Kan YW, Ma Q. Accelerated ovarian failure induced by 4-vinyl cyclohexene diepoxide in Nrf2 null mice. Mol Cell Biol. 2006;26:940–54. 16428448 10.1128/MCB.26.3.940-954.2006 PMC1347017

[63] Noda Y, Ota K, Shirasawa T, Shimizu T. Copper/zinc superoxide dismutase insufficiency impairs progesterone secretion and fertility in female mice. Biol Reprod. 2012;86:11–6. 10.1095/biolreprod.111.092999 21900685

[64] Huang B, Lu J, Ding C, Zou Q, Wang W, Li H. Exosomes derived from human adipose mesenchymal stem cells improve ovary function of premature ovarian insufficiency by targeting SMAD. Stem Cell Res Ther. 2018;9:1–12. 30092819 10.1186/s13287-018-0953-7 PMC6085638

[65] Ling L, Feng X, Wei T, Wang Y, Wang Y, Wang Z, et al. Human amnion-derived mesenchymal stem cell (hAD-MSC) transplantation improves ovarian function in rats with premature ovarian insufficiency (POI) at least partly through a paracrine mechanism. Stem Cell Res Ther. 2019;10:46. 10.1186/s13287-019-1136-x. 30683144 10.1186/s13287-019-1136-x PMC6347748

